# Castor Oil Plant (*Ricinus communis* L.) Leaves Improve Dexamethasone-Induced Muscle Atrophy *via* Nrf2 Activation

**DOI:** 10.3389/fphar.2022.891762

**Published:** 2022-07-05

**Authors:** Hyunjung Lee, Young In Kim, Min Jung Kim, Jeong-Hoon Hahm, Hyo Deok Seo, Tae Youl Ha, Chang Hwa Jung, Jiyun Ahn

**Affiliations:** ^1^ Aging and Metabolism Research Group, Korea Food Research Institute, Wanju-gun, South Korea; ^2^ Healthcare Research Group, Korea Food Research Institute, Wanju-gun, South Korea; ^3^ Department of Food Biotechnology, University of Science and Technology, Daejeon-si, South Korea

**Keywords:** *Ricinus communis* L., castor oil plant leaf, muscle atrophy, oxidative stress, rutin

## Abstract

Skeletal muscle atrophy is characterized by reduced muscle function and size. Oxidative stress contributes to muscle atrophy but can be treated with antioxidants. This study investigated the antioxidant activity of a castor oil plant leaf (*Ricinus communis* L.) extract (RC) and its effects on muscle atrophy. Rutin was identified as the major compound among the thirty compounds identified in RC *via* LC-MS/MS and was found to inhibit dexamethasone (DEX)-induced muscle atrophy and mitochondrial oxidative stress. Rutin-rich RC showed DPPH and ABTS radical scavenging activities and efficiently reduced the DEX-induced myotube atrophy and mitochondrial oxidative damage in C2C12 cells. RC supplementation prevented the loss of muscle function and muscle mass in DEX-administered mice and ameliorated DEX-induced oxidative stress *via* Nrf2 signaling. Taken together, both RC and rutin ameliorated muscle atrophy and helped in maintaining redox homeostasis; hence, rutin-rich RC could be a promising functional food that is beneficial for muscle health.

## 1 Introduction

Sarcopenia is characterized by the loss of muscle mass and a subsequent reduction in muscle function and is associated with reduced quality of life. The primary cause of muscle atrophy is aging. However, immobilization, prolonged bed rest, denervation, inflammation, and cachexia can also induce muscle atrophy (Walston, 2012). As the prevention or treatment of muscle atrophy remains unresolved, the development of therapeutic strategies to ameliorate muscle atrophy is imperative.

Oxidative stress contributes to the initiation and development of age-related sarcopenia (Fulle et al., 2004). Increased reactive oxygen species (ROS) production contributes to muscle atrophy due to disuse (Powers et al., 2012), although it is still debatable whether ROS is the cause or consequence of muscle atrophy. The production of ROS is increased in inactivity-induced muscle atrophy, and treatment with antioxidants has been found to be effective against this type of muscle wasting (Kondo et al., 1991). Although the mechanism of how inactivity increases ROS production is still unknown, it has been suggested that immobilization increases oxidative stress in skeletal muscles due to decreased antioxidant capacity and increased production of ROS (Kavazis et al., 2009). Increased ROS levels may lead to development of muscle atrophy *via* the acceleration of proteolysis and the suppression of protein synthesis (Powers et al., 2011a). Mitochondria are the site of ROS production, and disuse-induced oxidative stress has been linked to mitochondrial dysfunction (Powers et al., 2011b).

Dexamethasone (DEX), a synthesized glucocorticoid, is widely used clinically to treat inflammation and autoimmune diseases. However, the extended use of DEX evokes muscle wasting by reducing protein synthesis, *via* IGF-1 and mTOR, and promoting protein breakdown *via* the Forkhead box O (FOXO) one and 3-activated ubiquitin-proteosome system (Sandri et al., 2004). The expression of two muscle atrophy-related E3 ubiquitin ligases, muscle atrophy F-box (MAFbx/Atrogin-1) and muscle RING-finger protein-1 (MuRF1), is increased in DEX-induced muscle atrophy (Bodine et al., 2001). Human and animal studies have demonstrated that chronic corticosteroid treatment disrupts the mitochondrial oxidative capacity, leading to oxidative damage to DNA. Thus, eliminating ROS or promoting mitochondrial function may have a protective effect against muscle atrophy (Liu et al., 2014).

The castor oil plant leaf (*Ricinus communis* L.), also called castor bean, belongs to the Euphorbiaceae family. Although it is native to Africa, it is currently found across all tropical regions (Jeyaseelan and Jashothan, 2012). Its seed oil is used in traditional medicine as a laxative, purgative, and lubricant. The seed extract of *R. communis* is reported to have various pharmacological activities, such as antifertility (Sandhyakumary et al., 2003), insecticidal (Sharma et al., 1991), antitumor (Lin and Liu, 1986), and antinociceptive (Okwuasaba et al., 1991) effects. However, castor seeds are toxic and contain three kinds of toxins, namely ricin, ricinine, and lecithin. Among them, ricin is extremely toxic and as little as 500 μg can be lethal for an adult human (Moshiri et al., 2016). The leaves of *R. communis* L, however, are edible and consumed as cooked vegetable. The methanol extracts of castor leaves have been reported to exert antioxidant activity (Nemudzivhadi and Masoko, 2014). Additionally, six compounds—gallic acid, quercetin, gentisic acid, rutin, epicatechin, and ellagic acid—with different pharmacological activities have been isolated from castor leaves (Singh et al., 2009). Among them, rutin has been shown to exert therapeutic potential for cancer, diabetes, and hypertension because of its strong antioxidant property (Sharma et al., 2013). Dietary rutin is metabolized to phenol derivatives including 3,4-dihydroxyphenylacetic acid, 3,4-dihydroxytoluene, 3-hydroxyphenylacetic acid, and 4-hydroxy-3-methoxyphenylacetic acid, and quercetin by colonic microbiota (Pashikanti et al., 2010). However, the effects of *R. communis* L. leaf extract (RC) or rutin on muscle atrophy has not yet been investigated.

This study aimed to evaluate the antioxidant and anti-muscle wasting effects of castor oil plant leaf extract. First, we performed LC-MS/MS analysis to identify the major compounds of RC, and suggested rutin as one of its functional compounds. Then, we measured the antioxidant activity of RC and the effect of RC on DEX-induced oxidative stress and myotube atrophy was examined in C2C12 cells. In addition, the effect of RC supplementation on DEX-induced oxidative damage and muscle wasting were measured in mice.

## 2 Material and Methods

### 2.1 Materials

Rutin, 1,1-diphenyl-2-picrylhydrazyl (DPPH), 2,2′-azino-bis (3-ethylbenzothiazoline-6-sulfonic acid) (ABTS), 3-[4,5-dimethylthiazol-2-yl]-2,5-diphenyltetrazolium bromide (MTT), dimethyl sulfoxide (DMSO), MG132, Tris, sucrose, MgCl_2_, EDTA, ATP, and DTT were purchased from Sigma Aldrich (St. Louis, MO, United States). SUC-LLVY-AMC was obtained from Enzo Life Sciences (New York, NY, United States), and Tissue-Tek® optimum cutting temperature Compound was purchased from Sakura Finetek (Torrance, CA, United States). Dulbecco’s modified Eagle’s medium (DMEM) and fetal bovine serum (FBS) were supplied by HyClone^TM^ (Logan, UT, United States). Horse serum and penicillin-streptomycin were obtained from Gibco BRL (Grand Island, NY, United States). Protease and phosphatase inhibitors and RIPA buffer were purchased from Thermo Fisher Scientific (Rockford, IL, United States). Antibodies against T-MHC, MHCⅠ, MHCⅡa, and MHCⅡb were obtained from the Developmental Studies Hybridoma Bank, University of Iowa (Iowa City, IA, United States). Antibodies against Atrogin-1(Fbx32), MuRF1, p70 S6 Kinase, and GCLC were purchased from Abcam (Cambridge, MA, United States). Antibodies against Phospho-mTOR (Ser2448), mTOR, Phospho-4E-BP1 (Thr37/46), 4E-BP1, Phospho-AKT (Ser473), AKT, Phospho-p70 S6 Kinase (Thr389), Phospho-FOXO3a (Ser294), and FOXO3a were obtained from Cell Signaling Technology (Beverly, MA, United States). Anti-puromycin antibodies were obtained from Millipore (Billerica, MA, United States). Anti- Nuclear factor (erythroid-derived 2)-like 2 (Nrf2), heme oxygenase-1 (HMOX1), and NAD(P)H: quinone acceptor oxidoreductase 1 (NQO1) were obtained from Novus Biologicals (Littleton, CO, United States). GAPDH, β-actin antibodies, and horseradish peroxidase-conjugated secondary antibodies were purchased from Santa Cruz Biotechnology (Dallas, TX, United States).

### 2.2 Preparation of RC

The dried leaves of *R. communis* L. were ground and extracted with 50% ethanol at 80 °C for 3 h using a Soxhlet extractor. The extract was concentrated using a rotary vacuum evaporator (Büchi Labortechnik AG, Flawil, Switzerland) and then freeze-dried. The extraction yield was 24.2%.

### 2.3 UHPLC-ESI-QTOF-MS and HPLC Analysis

An Acquity UPLC system (Waters Corporation, Miliford, MA, United States) coupled to a Waters SYNAPT G2-Si mass spectrometer (Waters Corp, Manchester, United Kingdom) was used for UHPLC-QTOF MS analysis. The sample analytes were separated on an ACQUITY UPLC BEH C18 (2.1 × 100 mm, 1.7 μm) column, with 0.1% formic acid in water (solvent A) and acetonitrile (solvent B). The elution conditions were as follows: initial condition of 97% A, 0–17 min with 97-20% A, 17–18 min with 20% A, and returning to 98% A with a 2 min re-equilibration step. The flow rate was set at 0.5 ml/min, and the injection volume was 5 μl. The mass spectrometer data were acquired in both positive and negative ion modes. The electrospray ionization (ESI) source parameters were as follows: TOF MS scan range m/z, 50–1,000; desolvation temperature, 400°C; desolvation gas flow, 900 L/h; source temperature, 110°C; cone energy, 40 V; collision energy ramp, 15–45 V; capillary voltage, 3.0 kV in positive ion mode and 0.5 kV in negative ion mode. Raw data were acquired and processed with the Progenesis QI software (Nonlinear Dynamic, Newcastle upon Tyne, United Kingdom).

These HPLC conditions were used for the quantitative analysis of rutin content. RC (50 mg/ml) was dissolved in methanol and filtered through a 0.2 μm syringe filter prior to HPLC analysis. The HPLC system (e2695, Waters, Milford, MA, United States) was equipped with a binary gradient system with variable UV/vis detector (2489, Waters, Milford, MA, United States). Approximately, 10 μl of RC was injected into a C18 reverse phase column (YMC-Pack ODS-AM, 250 × 4.6 mm D, S-5 μm, 12 nm, YMC, Kyoto, Japan). The binary mobile phase consisted of solvent A (H_2_O containing 0.1% formic acid) and solvent B (acetonitrile containing 0.1% formic acid). The gradient program for HPLC analysis was initiated with 100:0 solvent A/solvent B; 0–10 min, 100%–85% solvent A; 10–30 min, 75% solvent A; 30–40 min, 15% solvent A; 40–55 min, 10% solvent A; 55–60 min, 0% solvent A. The flow rate was 1 ml/min, and column temperature was maintained at 35°C throughout the run. The compounds were detected at 280 nm.

### 2.4 Antioxidant Activity Measurement

The antiradical activity of *R. communis* L. was determined using the free radical DPPH, after minor modification of the reported method (Da Porto et al., 2000). Equal volumes of different concentrations of RC (0.5, 1, 2.5, 5, and 10 μg/ml) and 100% ethanol solution of DPPH (0.2 mM) were mixed. The mixtures were shaken vigorously and incubated at 27°C for 30 min in the dark. Absorbance was then measured at 514 nm using a microplate reader (Infinite M200 PRO, Tecan, Männedorf, Switzerland). The percentage of scavenging activity against DPPH radical was calculated thereafter. The ABTS radical was produced by mixing equal volumes of ABTS (7.4 mM) and potassium persulfate (2.6 mM) and incubating the mixture for 12–16 h at 27°C in the dark; then, the mixture was further diluted with PBS to adjust the absorbance at 734 nm to 0.7. For the ABTS assay, 198 μl of ABTS radical solution and 2 μl of various concentrations of RC extract (0.5, 1, 2.5, 5, and 10 μg/ml) were mixed and incubated for 2 h at 27°C, then the absorbance was measured at 734 nm. The scavenging activity percentage against ABTS radical was calculated subsequently.

The intracellular levels of ROS in C2C12 (ATCC, Manassas, VA, United States) cells were detected with the 2′,7′- dichlorofluorescein diacetate (DCF-DA) fluorescence probe (Molecular Probes, Eugene, OR, United States). C2C12 cells were seeded at a density of 2 × 10^5^/well in a 6-well plate; after 24 h, cells were co-treated with RC (1 and 2.5 μg/ml) or rutin (0.1 and 0.25 μM) and 5 μM DEX for 24 h. To measure intracellular ROS accumulation, the cells were treated with 10 μM DCF-DA and incubated for 30 min at 37°C. After washing twice with PBS, the fluorescence of the cells was measured at 480/530 nm with a microplate reader (Infinite M200 PRO, Tecan, Männedorf, Switzerland). The fluorescent DCF-DA was observed under a fluorescence inverted microscope IX71 (Olympus Life Science, Tokyo, Japan). The levels of oxidative stress markers were measured using commercial kits. The superoxide dismutase activity assay kit, catalase activity assay kit, glutathione assay kit, lipid peroxidation (MDA) assay kit, and 8-hydroxy 2 deoxyguanosine ELISA kit were purchased from Abcam (Cambridge, United Kingdom). The NADPH oxidase ELISA kit was purchased from MyBioSource (San Diego, CA, United States).

### 2.5 C2C12 Cell Culture and Myotube Differentiation

C2C12 cells were maintained in DMEM high-glucose medium, containing 10% fetal bovine serum and 1% penicillin-streptomycin, under 5% CO_2_ at 37°C. For differentiation, confluent cells were exposed to a differentiation medium containing high-glucose DMEM supplemented with 2% horse serum. After 4 days of differentiation, the RC extract (1 and 2.5 μg/ml) or rutin (0.1 and 0.25 μM) were added to the differentiated myotubes and incubated for 24 h, along with 5 μM DEX treatment.

### 2.6 Measurement of Cell Viability

Cell viability was measured using the MTT assay. C2C12 cells were seeded at a density of 2 × 10^4^ cells/well in a 96-well plate. After 24 h, the cells were treated with RC (0.5, 1, 2.5, 5, and 10 μg/ml) or rutin (0.1, 0.25, 0.5, and 1 μM) for an additional 24 h. To measure cell viability, 20 μl of MTT solution (5 mg/ml) was added to each well and the cells were incubated for 4 h at 37°C (in the dark). The supernatants were subsequently removed, and 200 μl DMSO was added to each well to dissolve the formazan crystals. Absorbance was measured at 570 nm.

### 2.7 Immunofluorescence Assay

The differentiated C2C12 myotubes were fixed in 4% formaldehyde for 15 min, permeabilized with 0.1% saponin in PBS, and blocked with 3% bovine serum albumin in PBS. The cells were stained with total MHC antibody, followed by an Alexa Fluor 488-conjugated secondary antibody (Cell Signaling Biotechnology) and 4ʹ,6-diamidino-2-phenylindole (DAPI, Molecular Probes) in PBS. Images were captured using a fluorescence microscope (Olympus, Toyo, Japan) and analyzed using ImageJ (NIH, Bethesda, MD, United States). Fusion index was calculated as the percentage of the number of nuclei incorporated into myotubes to the total number of nuclei.

For measuring the cross-sectional area (CSA), the gastrocnemius muscles harvested from RC-treated mice were mounted in OCT-embedded blocks and stored at 80°C. The tissue block was cut into 7 μm sections with a low temperature cryo-microtome (CM 1850; Leica Microsystems, Wetzlar, Germany) and placed on glass slides. Tissue sections were fixed in ice-cold 20% acetone for 20 min and blocked with 10% FBS in PBS at room temperature for 1 h. The tissues were then incubated overnight at 4°C with anti-laminin antibody, conjugated with Alexa Fluor 488 (NB-300, Novus biologicals). To measure muscle type transition, muscle tissue sections were incubated with MHC1 (BA-F8, DSHB, Iowa City, IA, United States), MHCIIA (SC-71-C, DSHB), and MHCIIB (BF-F3, DSHB) in 1% BSA at 4°C overnight, followed by a 1 h incubation with rabbit anti-mouse IgG2b DyLight 405 (NBP1-72922, Novus biologicals), Alexa Fluor 488 goat anti-mouse IgG (A21121, Invitrogen, Carlsbad, CA, United States), and Alexa Fluor 594 goat anti-mouse IgM (A21044, Invitrogen), respectively. After staining, the tissues were washed twice with PBS and mounted with Fluoroshield (F6182, Sigma Aldrich). Images of stained tissues were acquired using a confocal microscope (Digital Eclipse C1 plus, Nikon, Tokyo, Japan).

### 2.8 Oxygen Consumption Rate (OCR) Measurement

Mitochondrial OCR was measured using the Seahorse XF24 extracellular Flux Analyzer (Agilent Technologies, Santa Clara, CA, United States). C2C12 cells were seeded in XF-24 cell culture microplates and either left untreated or treated with RC (1, 2.5 μg/ml) or rutin (0.1, 0.25 μM) and 5 μM DEX for 24 h. Afterward, the cell medium was replaced with assay medium containing 25 mM glucose, 1 mM pyruvate, and 1 mM glutamine, and the cells were transferred to a CO_2_-free incubator and maintained at 37°C for 1 h before the assay. The injection ports were loaded with 1.5 μM oligomycin A, 4 μM carbonyl cyanide-4-(trifluoromethoxy) phenylhydrazone (FCCP), and 1 μM antimycin A/rotenone. Results were analyzed using the Wave Controller Software (Agilent, CA, United States).

### 2.9 Animal Experiments

Seven-week-old male C57BL/6 mice were purchased from Orient Bio Inc (Seongnam, Korea). Animal studies were conducted in accordance with institutional and national guidelines, and all experimental procedures were approved by the Korea Food Research Institute (KFRI-IACUC, KFRI-M-19030). After 1 week of acclimatization, thirty mice were randomly divided into three groups (*n* = 10/group): group I, the vehicle-control group (CTL group); group II, the dexamethasone-injected group (DEX group); and group III, the DEX-injected and RC-supplemented group (RC group). Mice were fed experimental diets based on AIN-93M, with or without 0.1% RC, for 8 weeks. During the last 3 weeks, DEX was administered by intraperitoneal (i.p.) injection at 25 mg/kg for 7 days followed by 5 mg/kg for 14 days to induce muscle atrophy. After treatment, all mice were anesthetized with 5% isoflurane and sacrificed; then, their muscles were harvested and weighed.

### 2.10 Measurement of Muscle Mass and Performance

Body composition of each mouse was determined with Dual Energy X-ray Absorptiometry (DXA, InAlyzer, Medikors, Korea). Muscle grip strength was measured using the grip strength meter and a grid bar (Bioseb, Chaville, France). Mice grasped the grid with their forelimb, and the mean of five consecutive measurements for each animal was calculated. For treadmill measurements, after 2 days of training, the total running distance and running time were measured on a rodent treadmill (Ugo Basile, Varese, Italy). The incline was 10%. Mice were placed on a treadmill with a warm-up speed of 10 m/min and a 0.1 mA shock for 20 min. Following warm up, speed was increased 2 m/min every 2 min until the mouse remained on the shock grid for 10 s. The maximal time did not exceed 30 min for the test.

### 2.11 Measurement of Protein Synthesis (Surface Sensing of Translation, SUnSET) and Proteasome Activity

We used the non-isotopic SUnSET technique to measure *in-vitro* changes in protein synthesis, as reported previously (Lee et al., 2021). Briefly, C2C12 cells were differentiated and treated as described above, and 1 μg/ml puromycin was added to the culture medium at the last 1 h before harvest. The total protein fraction was isolated with RIPA buffer, and the expression of puromycin was estimated *via* western blotting.

To measure proteasome activity, the quadricep muscles were homogenized in lysis buffer containing 50 mM Tris, 250 mM sucrose, 5 mM MgCl_2_, 0.5 mM EDTA, 2 mM ATP, and 1 mM DTT, at pH 7.5. After centrifugation at 12,000 × *g* and 4°C for 30 min, the supernatants were immediately used for the measurement of chymotrypsin-like activity. The supernatant was incubated with 100 μM fluorescently tagged substrate, SUC-LLVY-AMC, for 1 h at 37°C in the absence or presence of 200 μM MG132, the specific proteasomal inhibitor. Fluorescence was detected using a fluorometric microplate reader at an excitation wavelength of 390 nm and emission wavelength of 460 nm. Proteasome activity was expressed as a percentage relative to that of the CTL group.

### 2.12 Quantitative Reverse Transcription-PCR (qRT-PCR) Analysis

Total RNA was extracted using NucleoSpin RNA II (Macherey-Nagel, Düren, Germany) for cells and the RNeasy Fibrous Tissue Mini kit (Qiagen, Germantown, MD, United States) for animal tissues. cDNA was synthesized using the ReverTra Ace qPCR RT Master Mix (Toyobo, Osaka, Japan). qRT-PCR was performed with the SYBR Green Master Mix (Toyobo Osaka, Japan) using a Vii7 Real-Time PCR System (Applied Biosystems, Foster City, CA, United States). Primer sequences are listed in Supplementary Table S1. The qPCR data was analyzed by 2^−ΔΔCT^ method (Livak and Schmittgen, 2001) and the relative mRNA levels were determined after normalization to the reference gene mRNA levels.

### 2.13 Western Blot Analysis

Total protein was isolated using RIPA lysis buffer (Thermo Fisher Scientific, Waltham, MA, United States). Nuclear and cytosolic fractions were prepared with the NE-PER™ Nuclear and Cytoplasmic Extraction Reagents kit (Thermo Fisher Scientific). Protein concentration was determined with an enhanced BCA protein assay kit (Thermo Fisher Scientific). Proteins (20 μg) were resolved with SDS-PAGE and transferred onto polyvinylidene difluoride membranes (Bio-Rad, Hercules, CA, United States). The membranes were blocked with 5% skim milk in TBST and incubated with primary antibodies overnight at 4°C. After washing several times with TBST buffer, the membranes were incubated with the appropriate HRP conjugated secondary antibody. Protein bands were visualized using a chemiluminescence reagent (Amersham Pharmacia Biotech, Piscataway, NJ, United States).

### 2.14 Statistical Analysis

Data are expressed as mean ± SEM for *in vivo* studies and as mean ± standard deviation (SD) for *in vitro* studies. Data were statistically analyzed using the GraphPad Prism 9 software (San Diego, CA, United States). One-way analysis of variance (ANOVA) was used to compare quantitative data across groups, followed by the Bonferroni post-hoc test. *p* < 0.05 indicated statistical significance.

## 3 Results

### 3.1 LC/MS-qTOF Analysis Identified Rutin as the Major Compound in RC

**FIGURE 1:**
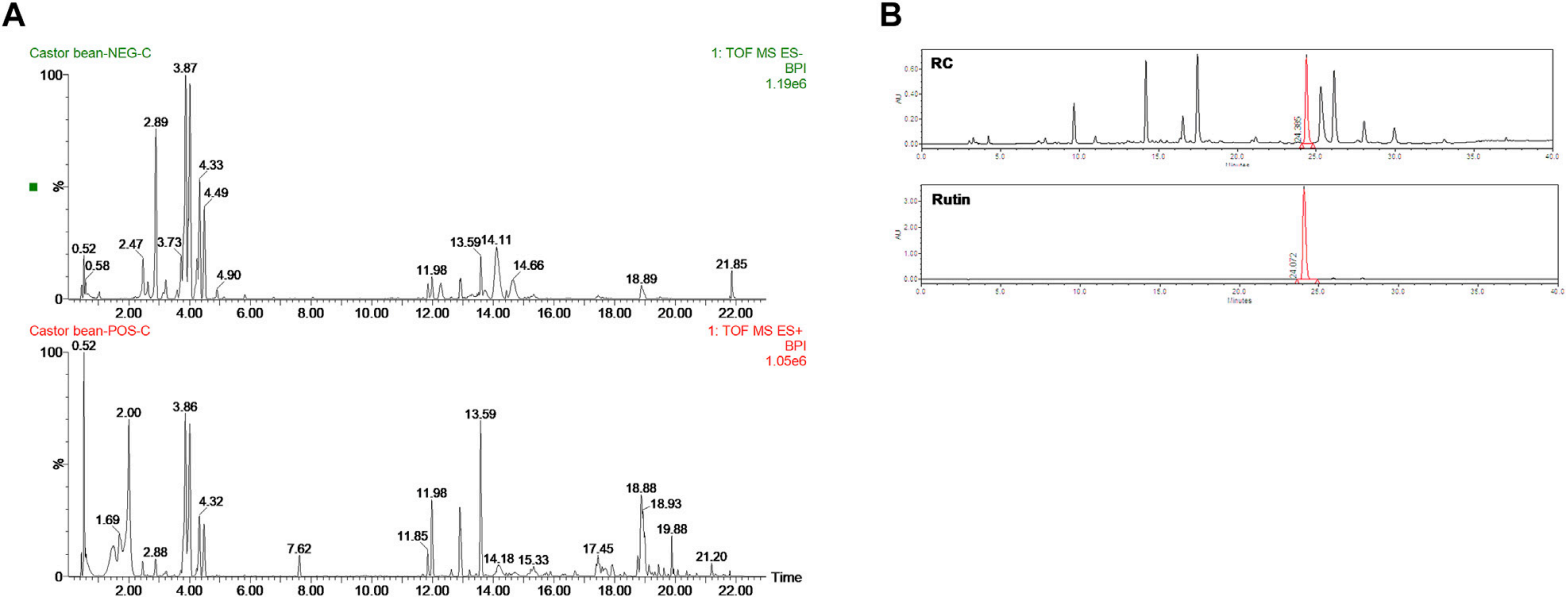
UHPLC-QTOF-MS analysis **(A)** in negative (top) and positive (bottom) modes and HPLC analysis **(B)** for the identification of RC components.

To determine the composition of RC, UHPLC-QTOF-MS analysis was performed using both positive and negative ion modes (Figure 1A). As shown in Table 1, 30 tentative compounds were identified in RC. We confirmed the presence of kaempferol 3-O glucoside, kaempferol-3-rutinoside, ellagic acid, and rutin in RC. Among them, rutin showed the highest intensity in the negative ion mode. Subsequently, we measured rutin content in RC, using HPLC analysis, and found it to be 159.5 mg/100 g dry weight of extract (Figure 1B).

**TABLE 1:** The list of identified in RC by LC-MS negative and positive mode analysis.

Putative Identification	Molecular Formula	Ion Mode	Exact Mass	Actual Mass	Mass Error (ppm)	RT (min)	Intensity
Hydroxybenzoic acid	C_7_H_6_O_3_	[M-H]^-^	137.0239	137.0223	1.6	2.12	26.1
Gentistic acid	C_7_H_6_O_4_	[M-H]^-^	153.0188	153.017	1.8	1.58	62.7
O-coumaric acid	C_9_H_8_O_3_	[M-H]^-^	163.0395	163.038	1.5	2.52	93.4
Gallic acid	C_7_H_6_O_5_	[M-H]^-^	169.0137	169.0126	1.1	1.01	3003
Caffeic acid	C_9_H_8_O_4_	[M-H]^-^	179.0344	179.0332	1.2	3.08	371
Quinic acid	C_7_H_12_O_6_	[M-H]^-^	191.0556	191.0549	0.7	0.52	1064
Ethyl gallate	C_9_H_10_O_5_	[M-H]^-^	197.045	197.0438	1.2	3.56	2150.9
Narigenin	C_15_H_12_O_5_	[M-H]^-^	271.0606	271.058	2.6	6.55	20.3
Kaempferol	C_15_H_10_O_6_	[M-H]^-^	285.0399	285.039	0.9	6.74	1208.3
Eriodictyol	C_15_H_12_O_6_	[M-H]^-^	287.0556	287.0529	2.7	4.04	56.7
Ricinoleic acid	C_18_H_34_O_3_	[M-H]^-^	297.243	297.2415	1.5	13.81	61.4
Ellagic acid	C_14_H_6_O_8_	[M-H]^-^	300.9984	300.9984	0.0	3.68	12127.7
Quercetin	C_15_H_10_O_7_	[M-H]^-^	301.0348	301.0339	0.9	5.8	2834.2
Isorhamnetin	C_16_H_12_O_7_	[M-H]^-^	315.0505	315.0489	1.6	5.96	76.7
Protocatechuic acid 4-O-glucoside	C_13_H_16_O_9_	[M-H]^-^	315.0716	315.0693	2.3	1.43	42.7
Myricetin	C_15_H_10_O_8_	[M-H]^-^	317.0297	317.0283	1.4	4.23	173.9
Digallic acid	C_14_H_10_O_9_	[M-H]^-^	321.0247	321.0213	3.4	3.48	26.9
Galloyl glucose	C_13_H_16_O_10_	[M-H]^-^	331.0665	331.0657	0.8	0.57	3218.7
Chlorogenic acid	C_16_H_18_O_9_	[M-H]^-^	353.0873	353.0834	3.9	2.33	7.7
Caffeoylquinic acid	C_16_H_18_O_9_	[M-H]^-^	353.0873	353.0858	1.5	4.78	39.4
Apignin-7-glucoside	C_21_H_20_O_10_	[M-H]^-^	431.0978	431.096	1.8	5.29	62
Naringenin 7-O-glucoside	C_21_H_22_O_10_	[M-H]^-^	433.1135	433.1118	1.7	4.73	60.6
Kaempferol-3-glucoside	C_21_H_20_O_11_	[M-H]^-^	447.0927	447.0922	0.5	4.46	22866.5
Isorhamnetin 3-O-glucoside	C_22_H_22_O_12_	[M-H]^-^	477.1033	477.1003	3.0	3.64	99.1
Kaempferol 3-O-acetyl-glucoside	C_23_H_22_O_12_	[M-H]^-^	489.1033	489.1001	3.2	5.23	225.9
Dicaffeoylquinic acid	C_25_H_24_O_12_	[M-H]^-^	515.119	515.1173	1.7	4.78	258.1
Kaempferol 3-O-(6″-malonyl-glucoside)	C_24_H_22_O_14_	[M-H]^-^	533.0931	533.0884	4.7	4.66	136.8
Kaempferol-3-rutinoside	C_27_H_30_O_15_	[M-H]^-^	593.1506	593.1504	0.2	4.3	30266.7
Rutin	C_27_H_30_O_16_	[M-H]^-^	609.1456	609.1453	0.3	3.84	50158.2
Coumarin	C_9_H_6_O_2_	[M + H] ^+^	147.0446	147.0419	2.7	2.53	50.2

### 3.2 Rutin Exerts Anti-Muscle Atrophy and Improves Mitochondrial Function in DEX-Treated C2C12 Myotubes

**FIGURE 2:**
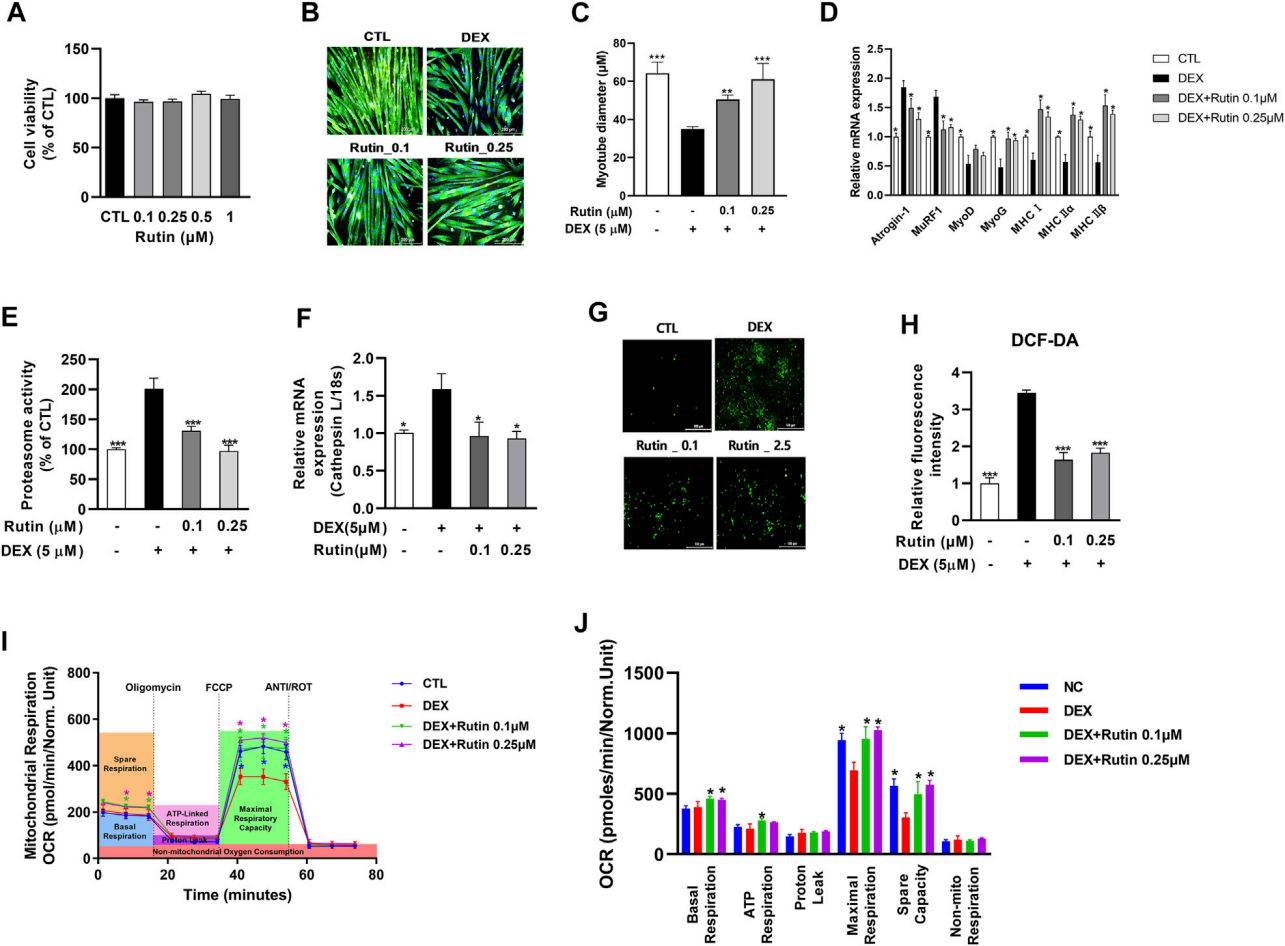
Effect of rutin on mitochondrial oxidative stress and myotube atrophy. Effect of rutin on cell viability **(A)**, dexamethasone (DEX)-induced myotube atrophy **(B)**, myotube diameter **(C)**, atrophy-related gene expression **(D)**, proteasome activity **(E)**, relative cathepsin L expression **(F)**, intracellular ROS accumulation **(G)**, and fluorescent intensity of DCF-DA **(H)** was measured. Mitochondrial OCR **(I)** and mitochondrial respirations **(J)** were examined by Seahorse XF24 analyzer. OCR was normalized to cell number. Data are shown as mean values ±SD. ∗*p* < 0.05, ∗∗*p* < 0.01, ∗∗∗*p* < 0.001 versus DEX.

Next, we examined whether rutin is a bioactive compound of RC and has preventive effects against muscle atrophy. First, we established that treatment with rutin did not affect cell viability at concentrations ≤1 μM (Figure 2A). To examine the effect of rutin on DEX-induced muscle atrophy, C2C12 myotubes were co-treated with 5 μM DEX and 0.1 or 0.25 μM rutin. Immunofluorescence staining of MHC revealed that rutin remarkably inhibited the DEX-induced myotube atrophy (Figure 2B). Measurement of the myotube diameter confirmed the anti-muscle atrophy activity of rutin (Figure 2C). qRT-PCR analysis revealed that rutin reduced the activity of muscle atrophy-related E3 ubiquitin ligases, such as Atrogin-1 and MuRF1, and increased the expression of myogenic regulatory factors, including MyoD and myogenin (MyoG), and MHC isoforms (Figure 2D). Of note, rutin also induced upregulation of MyoD and MyoG in C2C12 myoblasts in the absence of DEX (Supplementary Figure S1A). Exposure to DEX induced an increase in proteosome activity and led to the upregulation of cathepsin in C2C12 myotubes (Figures 2E,F). Rutin effectively reduced the proteasome activity and downregulated the mRNA expression of cathepsin L.

Elevated ROS levels can lead to muscle atrophy through the induction of atrogens, such as Atrogin-1 and MuRF1 (Rodney et al., 2016). We used the fluorescence of DCF-DA to determine the intracellular ROS levels. As shown in Figures 2G,H, ROS production was enhanced in DEX-treated C2C12 myotubes. Furthermore, we observed that rutin effectively decreased the DEX-induced intracellular ROS accumulation in C2C12 myotubes. As mitochondrial dysfunction has been shown to contribute to muscle atrophy *via* oxidative stress (Muller et al., 2007), we measured the mitochondrial oxygen consumption rate (OCR), using Seahorse XF24 Extracellular Flux Analyzer. Interestingly, treatment with rutin significantly increased the mitochondrial maximal respiration in C2C12 cells in the absence of DEX (Supplementary Figures S2A, S2B). Furthermore, the DEX-induced decrease in mitochondrial OCR was effectively recovered after co-treatment with rutin (Figure 2I). We observed that the basal, ATP-linked, and maximal respiration as well as the spare capacity were significantly increased in rutin-treated C2C12 cells (Figure 2J) (*p* < 0.05). These findings suggest that rutin inhibits DEX-induced muscle atrophy *via* antioxidant activity.

### 3.3 RC Attenuated the DEX-Induced Muscle Atrophy and Oxidative Stress in C2C12 Myotubes

**FIGURE 3:**
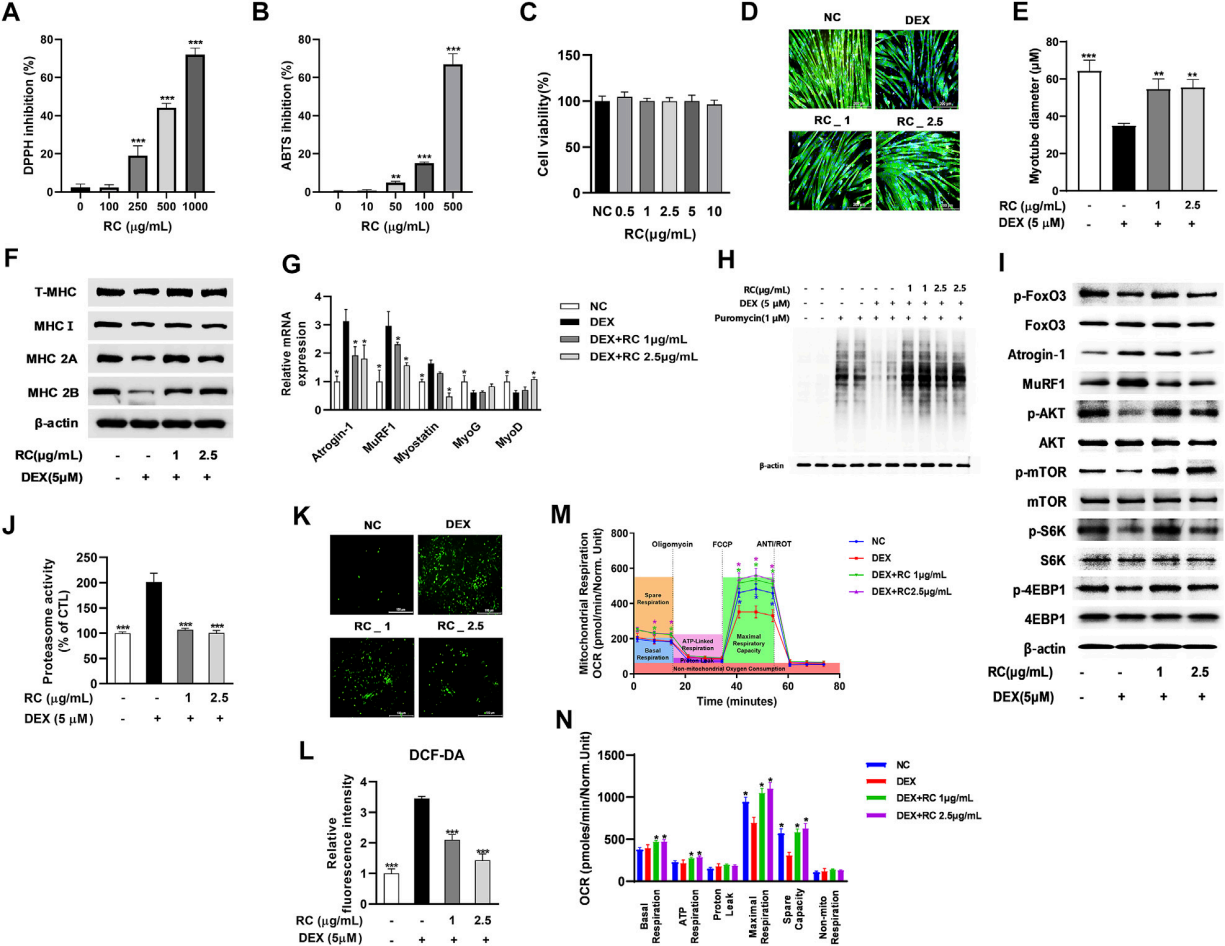
Antioxidant and anti-myotube atrophy effects of 50% ethanolic extract of the castor oil plant (*Ricinus communis* L.) leaves (RC). DPPH **(A)** and ABTS **(B)** inhibitory activities of RC. Data are shown as mean values ±SD. ∗∗*p* < 0.05, ∗∗∗*p* < 0.001 versus control. Effect of RC on cell viability **(C)** DEX-induced myotube atrophy **(D)**, myotube diameter **(E)**, MHC isoform expression **(F)**, atrophy- or myogenesis-related genes **(G)**, protein synthesis **(H)**, protein synthesis-related signaling **(I)**, proteasome activity **(J)**, intracellular ROS accumulation **(K)**, and fluorescence intensity of DCF-DA **(L)** was measured. Mitochondrial oxygen consumption rate (OCR) **(M)** and mitochondrial respiration **(N)** were examined by Seahorse XF24 analyzer. OCR was normalized to cell number. Data are shown as mean values ±SD. ∗*p* < 0.05, ∗∗*p* < 0.01, ∗∗∗*p* < 0.001 versus DEX.

To examine the antioxidant activity of RC, we measured its DPPH radical scavenging activity at concentrations of 100–1000 μg/mL. As shown in Figure 3A, RC dose-dependently inhibited DPPH radical production, and the IC_50_ was 719 μg/ml. Next, we measured the ABST radical scavenging activity of RC and observed that it also inhibited ABTS production in a dose-dependent manner, with the IC_50_ value of 719 μg/ml (Figure 2B). These results indicated that RC possesses strong antioxidant activity.

Next, we measured the effect of RC on DEX-induced myotube atrophy. RC showed no cytotoxicity at concentrations ≤10 μg/ml in C2C12 cells (Figure 3C). Immunofluorescent staining of MHC revealed that DEX triggered myotube atrophy with remarkably reduced myotube diameters, whereas RC protected against this DEX-induced decrease in myotube size (Figure 3D). The myotube diameter of the muscle samples decreased from 64.33 to 34.97 μm upon DEX treatment, while it increased to 50.55 μm after treatment with 1 μg/ml of RC and further to 61.20 μm upon treatment with 2.5 μg/ml of RC (Figure 3E). The results of western blot analysis revealed increased protein expression of MHC isoforms upon RC treatment (Figure 3F).

Further, we investigated the effect of RC on the mRNA expression of muscle atrophy- and myogenesis-related genes (Figure 3G). RC distinctly suppressed the DEX-induced upregulation of muscle-specific ubiquitin ligases, also known as atrophy related-genes (atrogens), such as Atrogin-1 and MuRF1. Moreover, RC treatment reduced the mRNA levels of myostatin in a dose-dependent manner. Interestingly, the levels of myogenesis-associated markers, such as MyoD and myogenin (MyoG), were significantly increased upon RC treatment (*p* < 0.05). Of note, RC also induced upregulation of MyoD and MyoG in C2C12 myoblasts in the absence of DEX (Supplementary Figure S1B). Finally, we performed SUnSET, a nonradioactive method to monitor protein synthesis, and found that the DEX-induced decrease in protein synthesis was recovered upon RC treatment (Figure 3H).

We next explored the molecular mechanism by which RC regulates proteostasis. FOXO3 is an important transcriptional regulator and induces the transcription of atrogens and cathepsin (Mammucari et al., 2007); our assays showed that the phosphorylation of FOXO3 was increased by RC. Consistent with mRNA levels, atrogen protein levels were also reduced after RC treatment (Figure 3I). The phosphorylation of mTOR, Akt, S6K, and 4-EBP1 was reduced by DEX, which in turn indicated that DEX inhibited protein synthesis. However, RC treatment reversed the phosphorylation of mTOR and its downstream targets. Measurement of the chymotrypsin-like activity of the 26S proteasome revealed that the elevated proteasome activity due to DEX was effectively reduced by RC (Figure 3J). These results suggested that RC effectively attenuated muscle protein degradation *via* ubiquitin- and proteosome-mediated muscle atrophy signaling.

Following investigation of the anti-muscle atrophy effect of RC, its antioxidant effect against DEX-induced oxidative stress was examined. As shown in Figure 3K, L treatment with RC significantly reduced the DEX-induced ROS production. To examine the effect of RC on DEX-induced mitochondrial dysfunction, we measured mitochondrial OCR in DEX- and DEX + RC-treated C2C12 cells (Figure 3M). As expected, the mitochondrial respiratory capacity was reduced by DEX, and this reduction was completely prevented by RC co-treatment. RC effectively recovered the basal and maximal mitochondrial and ATP respiration (Figure 3N). Treatment with RC only significantly increased the maximal respiratory capacity of C2C12 cells in the absence of DEX (Supplementary Figures S2C, S2D). These results together suggest that the antioxidant effect of RC contributes to the attenuation of myotube atrophy through an increase in mitochondrial activity and a decrease in protein degradation.

### 3.4 RC Protects C57BL/6 Mice From DEX-Induced Muscle Wasting

**FIGURE 4:**
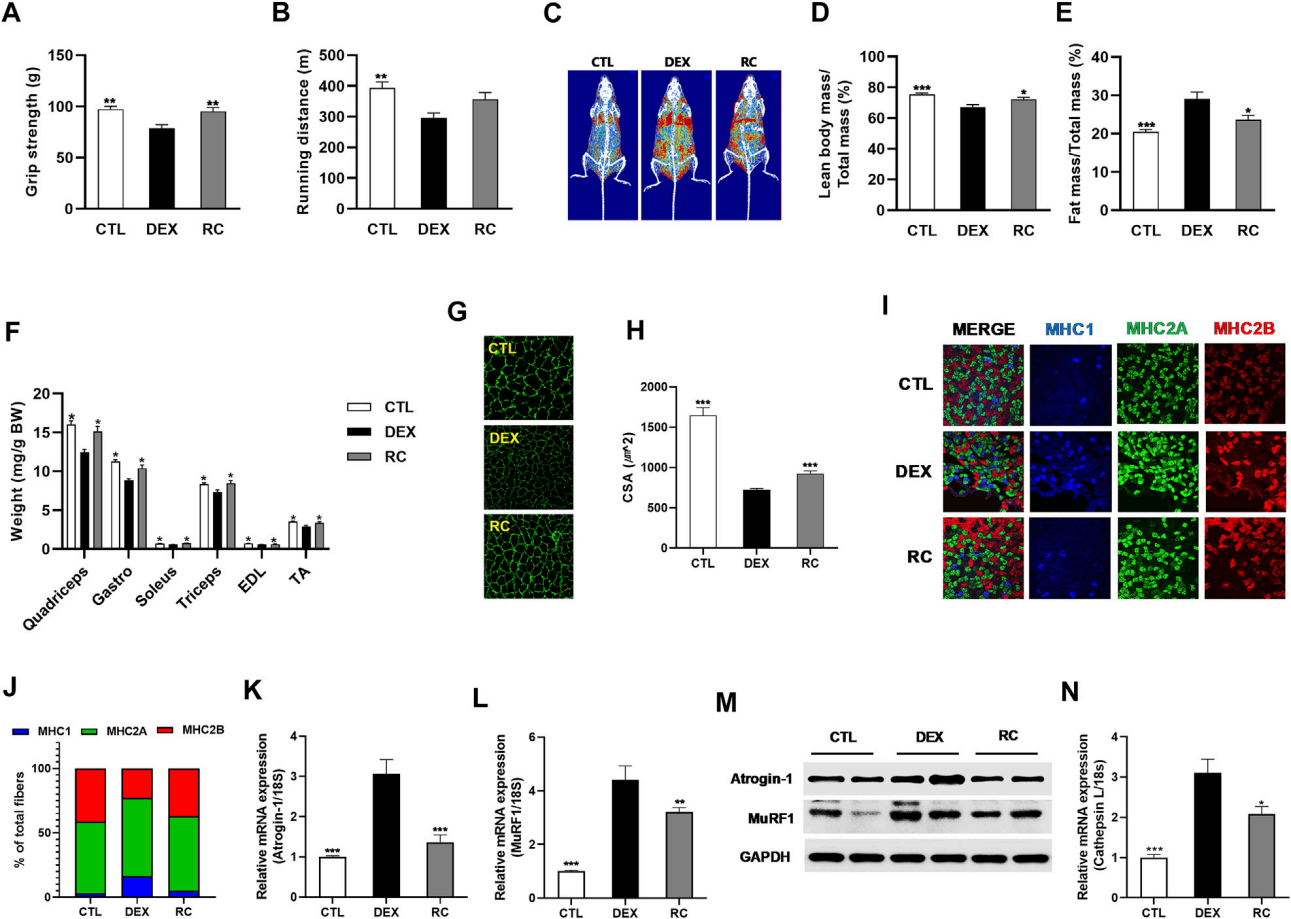
Anti-muscle wasting effect of RC. Muscle function **(A)**, grip strength; **(B)**, tread mill exercise and muscle mass **(C)**, representative DXA scan images; **(D)**, percentage of lean body mass; **(E)**, body fat percentage; **(F)**, forelimb and hindlimb muscle weights were normalized to the total body weight. Effect of RC on muscle cross-sectional area (CSA) **(G,H)** and muscle type transition **(I,J)** was studied. Effect of RC on mRNA **(K** and **L)** and protein **(M)** expression of atrogens is shown. Relative cathepsin L mRNA expression **(N)**. Data are shown as mean values ±SEM. ∗*p* < 0.05, ∗∗*p* < 0.01, ∗∗∗*p* < 0.001 versus DEX.

We examined whether supplementation of RC could ameliorate DEX-induced muscle atrophy *in vivo*. To this end, we fed C57BL/6 mice with an experimental diet containing 0.1% RC for 8 weeks. DEX was injected in the last 3 weeks to induce muscle atrophy and muscle dysfunction. After 8 weeks, measurement of grip strength and treadmill exercise capacity revealed impaired muscle function in the DEX group (Figures 4A,B). The DXA analysis revealed a decrease in lean body mass and an increase in fat mass in the DEX group (Figures 4C–E). Consistent with the decrease in lean body mass, the weights of quadriceps, gastrocnemius, soleus, triceps, EDL, and TA muscles were significantly reduced in the DEX group (Figure 4F) (*p* < 0.05). These data confirmed the induction of muscle atrophy by glucocorticoid administration. However, treatment with RC efficiently inhibited the DEX-induced damage to muscle function and loss of muscle mass (Figures 4A–F). Furthermore, the cross-section area (CSA) of the gastrocnemius muscle was increased upon RC treatment (Figures 4G,H). Overall, the decrease in muscle mass and muscle function due to DEX administration was reversed by RC supplementation.

Since glucocorticoid treatment primarily affects fast-twitch type II glycolytic muscle fibers (Schakman et al., 2008), we measured the fiber type composition of the gastrocnemius muscle, which is a heterogeneous tissue with predominant type II muscle. We observed that treatment with DEX induced a shift towards increased levels of type I muscle (Figures 4I,J). However, RC effectively reversed the DEX-induced muscle fiber transition. The DEX-induced increased expression of Atrogin-1 and MuRF1 was reduced by RC at both mRNA and protein levels (Figure 4K–M). We then measured the mRNA expression of cathepsin L and found that the DEX-induced upregulation of cathepsin L was significantly suppressed by RC (Figure 4N) (*p* < 0.05). Overall, these data indicate that RC effectively protects against DEX-induced muscle atrophy.

### 3.5 RC Reduces the Level of DEX-Induced Oxidative Stress in Muscle Tissues

**FIGURE 5:**
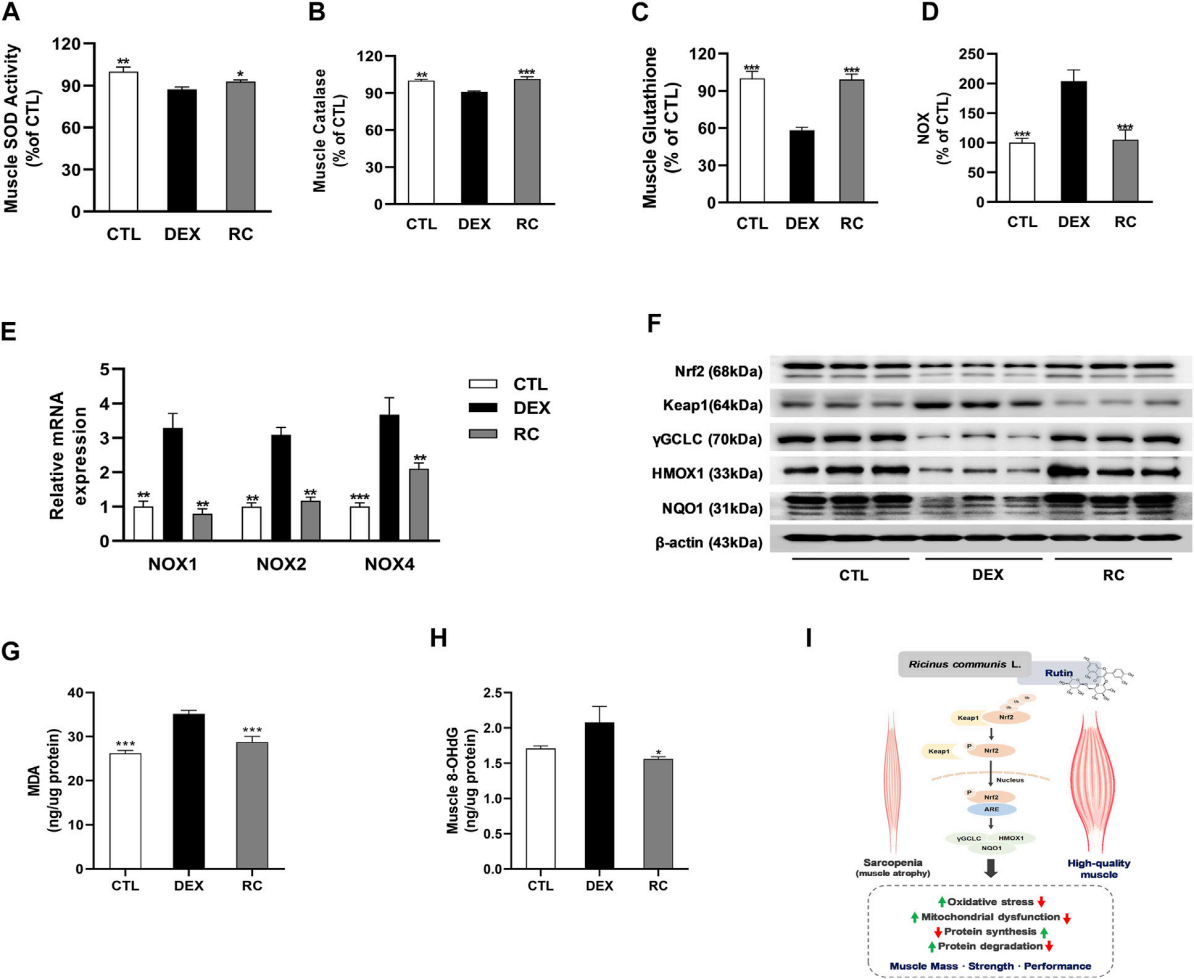
Effect of RC on oxidative stress in skeletal muscle atrophy. Muscle antioxidant activities, including superoxide dismutase (SOD, **(A)**, catalase **(B)**, and glutathione content **(C)**. Enzyme activity **(D)** and isoform mRNA expression **(E)** of NADPH oxidase (NOX) were measured. Western blot analysis of Nrf2 and its downstream targets were performed **(F)**. Malondialdehyde (MDA) for lipid peroxidation **(G)** and 8-hydroxy-2ʹ-deoxyguanosine (8-OHdG) for DNA damage **(H)** were measured. Data are shown as mean values ±SEM. **(I)** The potential mechanism of RC and rutin for the improvement of muscle atrophy. ∗*p* < 0.05, ∗∗*p* < 0.01, ∗∗∗*p* < 0.001 versus DEX.

To elucidate the potential involvement of antioxidant effect of RC in the attenuation of muscle wasting, we measured the levels of oxidative stress-associated markers in gastrocnemius muscles. First, we measured the activities of three major classes of enzymatic antioxidants, namely super oxide dismutase (SOD), catalase, and glutathione peroxidase (GPX), and found that the injection of glucocorticoids induced oxidative stress in muscle tissues (Figures 5A–C). However, supplementation with RC increased the activities of antioxidant enzymes and glutathione (GSH). DEX-treated mice exhibited an increase in NADPH oxidase (NOX) activity and upregulation of NOX1, NOX2, and NOX4 at the mRNA level when compared to that in the CTL group (Figures 5D,E). We observed that the DEX-induced increase in NOX activity and upregulation of NOX isoforms were significantly inhibited by RC (*p* < 0.001 and *p* < 0.01, respectively).

Administration of DEX reduced the protein expression of nuclear factor (erythroid-derived 2)-like 2 (Nrf2) and the Nrf2 targeting genes, namely, heme oxygenase-1 (HO-1), NAD(P)H: quinone acceptor oxidoreductase 1 (NQO1), and gamma glutamate cysteine ligase (γGCL), *via* the inhibition of Keap1 (Figure 5F); this decrease was noticeably reversed by RC supplementation. These results indicate that RC induces Nrf2 activation. Finally, we measured malondialdehyde (MDA) and 8-hydroxy two deoxyguanosin (8-OHdG) contents as markers of lipid oxidative damage and DNA oxidative damage, respectively (Figures 5G,H). Data showed that RC effectively reduced the DEX-induced elevation of MDA and 8-OHdG contents. Collectively, these findings indicate that RC reduces oxidative stress effectively in DEX-induced muscle atrophy *via* Nrf2 activation.

## 4 Discussion

In this study, we report the effect of RC on the C2C12 myotube atrophy and mouse muscle atrophy induced by DEX. Our results revealed that RC improved DEX-induced muscle atrophy *in vitro* and *in vivo*, as evidenced by increased muscle function and muscle mass, decreased atrogen levels, and improved proteostasis, which were associated with reduced oxidative stress *via* Nrf2 activation. Rutin, a functional polyphenol of RC, likely mediates the inhibitory effect of RC on muscle atrophy *via* its antioxidant activity.

The leaves of *R. communis* L. have been reported to exert various pharmacological activities, such as antifungal and antimicrobial effects (Panghal et al., 2011). The extracts of the aerial parts of *Ricinus communis* have also reported to show antioxidant activity (Iqbal et al., 2012). It has been reported that the ethanolic extracts of RC seeds and leaves have IC_50_ values of 77.17 ± 0.36 and 62.39 ± 0.18 μg/ml, respectively, in DPPH scavenging assays (Ihekuna et al., 2019). Studies using different solvents suggested that the antioxidant activity of the leaves of *R. communis* L. is strongest in the 80% methanol extraction. Our RC exerted DPPH and H_2_O_2_ inhibitory effects similar to those of the methanolic extract of *Ricinus communis* leaves reported in a previous study (Babu et al., 2017).

In the castor oil plant, kaempferol-3-O-β-D-xylopyranoside, kaempferol-3-O-β-D-glucopyranoside, kaempferol-3-O-β-rutinoside, quercetin-3-O-rutinoside, quercetin-3-O-β-D-xylopyranoside, quercetin, and rutin are the major flavonoids (Kumar, 2017). Using LC/MS analysis, we detected 30 peaks and identified rutin as the major compound in RC. Additionally, rutin is a functional component of the methanolic extract of *Ricinus communis* leaves (Babu et al., 2017). Rutin is a flavonoid well known for its antioxidant property. The antioxidant activity of flavonoid is determined by the total amount and configuration of the hydroxyl group (Burda and Oleszek, 2001). Rutin (quercetin-3-O-rutinose) is composed of one molecule of quercetin (3,3′,4′,5,7-pentahydroxyflavone) as aglycone and rutinose. Though aglycones show higher antioxidant effects than their corresponding glycosides (Plumb et al., 1999), rutin has slightly weaker antioxidant activity than quercetin (Mora et al., 1990). Addition of a catechol group on the B-ring dramatically decreased the antioxidant activity of rutin (Kessler et al., 2010). During oxidative stress, it is oxidized to rutin quinone and upregulates the Nrf2-mediated endogenous antioxidant response (Sthijns et al., 2017). Rutin has been shown to increase mitochondrial biogenesis and ATP synthesis in C2C12 myotubes (Chang et al., 2021) and reduce diet-induced obesity by causing an increase in muscle mitochondrial biogenesis in mice (Kim et al., 2015). Our *in vitro* data suggest that rutin mitigates the DEX-induced oxidative stress and reduces mitochondrial respiration in C2C12 myotubes. In animals, Vitamin E, a well-known anti-inflammatory agent, functions through the inhibition of NF-κB, thereby reducing proteolysis and muscle atrophy (Servais et al., 2007; Khor et al., 2014). Similarly, rutin attenuates lipopolysaccharide-induced inflammatory response in C2C12 cells (Liu et al., 2019). We found that rutin inhibited DEX-induced myotube atrophy *via* suppressing protein degradation.

Muscle atrophy can result from increased protein degradation and reduced protein synthesis. Thus, targeting proteostasis is a pivotal approach to combat muscle atrophy. The ubiquitin-proteasome system degrades myofibrillar proteins in muscle atrophy and is composed of ubiquitin ligases and the proteasome (Bilodeau et al., 2016). The two muscle-specific E3 ligases, Atrogin-1 and MuRF1, are involved in the ubiquitination of sarcomeric proteins (Lokireddy et al., 2012) and myosin heavy chain (Cohen et al., 2009), respectively. These two genes are markedly induced in various types of muscle atrophy (Gomes et al., 2001). Thus, Atrogin-1 and MuRF1 are biomarkers of accelerated proteolysis and muscle atrophy process. In muscles, activation of PI3K-AKT signaling promotes protein accumulation by suppressing the FOXO3 transcription factor, which increases proteolysis through controlling the expression of atrogens (Latres et al., 2005). AKT phosphorylates FOXO3 at three sites, including threonine 32, serine 253, and serine 315, and leads to inhibition of the transactivation activity of FOXO3 through nuclear export (Brunet et al., 1999). Our results showed that treatment with DEX distinctly inhibited protein synthesis. However, RC effectively recovered protein synthesis and induced the upregulation of Atrogin-1 and MuRF1. In this study, RC reversed the AKT-induced phosphorylation of FOXO3, which resulted in the inhibition of atrogen induction.

Studies of the molecular pathways involved in the loss of skeletal muscle mass and function have reported that dysregulation of redox balance is a major mechanism leading to muscle wasting. In an atrophic muscle, the balance shifts in favor of pro-oxidants and triggers oxidative stress. Human and animal studies have demonstrated that chronic corticosteroid treatment disrupts mitochondrial oxidative capacity and can lead to DNA oxidative damage (Mitsui et al., 2002). Myogenic cells contain antioxidant enzymes, including superoxide dismutase (SOD), catalase, glutathione peroxidase (GPX), γ-glutamylcysteine synthetase, and heme oxygenase-1 (HO-1) (Powers et al., 1994). These antioxidants are abundantly present in oxidative muscle fibers such as Type I and IIa/x, and their deficiency is indicative of oxidative stress. These enzymes not only scavenge excess ROS but also influence muscle cell function (Powers et al., 2011c). Thus, antioxidant enzymes are considered therapeutic targets for muscle diseases such as Duchenne muscular dystrophy (Petrillo et al., 2017). Here, we found that RC increased antioxidant enzyme activities in atrophic muscle tissues.

Mitochondrial dysfunction in various muscle atrophy models, such as disuse, diabetes, and aging, has been extensively investigated (Romanello et al., 2010; Romanello and Sandri, 2013). Improvement of mitochondrial function *via* the elimination of ROS or enhancement of mitochondrial biogenesis has been shown to protect against muscle atrophy (Liu et al., 2014). In this study, RC and its main active compound rutin effectively prohibited the occurrence of DEX-induced mitochondrial damage in C2C12 myotubes.

NOX is a multicomponent enzyme that catalyzes the reduction of O_2_ to O_2_^−^. In skeletal muscle, two isoforms, NOX2 and NOX4, are considered to be the major source of ROS (Sakellariou et al., 2014). We observed that administration of DEX induced the upregulation of NOX isoforms in skeletal muscles, and that this increase was effectively suppressed by RC. This suggests that RC effectively inhibits NOX-involved oxidative stress in DEX-induced muscle atrophy, as evidenced by reduced MDA and 8-OHdG levels.

Nrf2 is a leucine zipper transcription factor activated by the disassociation of oxidized Keap1. The promoters of HMOX1 and NQO1 contain antioxidant response elements (AREs), which form the binding site of Nrf2 (Ma, 2013). Additionally, Nrf2 activates the transcription of glutamate cysteine ligase (GCL), which is a rate-limiting enzyme in the biosynthesis of GSH and consists of the catalytic (GCLC) and modifier (GCLM) subunits. Thus, Nrf2 acts as the central transcriptional regulator in ARE-driven gene expression and maintains intracellular redox homeostasis in response to oxidative stress. Interestingly, Nrf2 KO mice and aged mice exhibit distinct similarities in intense sensitive responses to various stressors, suggesting that Nrf2/Keap1 redox signaling is involved in the aging process (Kensler et al., 2007). A previous study on the role of Nrf2/Keap1 signaling in aged skeletal muscle revealed increased Nrf2 and decreased Keap1 contents in active elderly subjects, and the opposite in sedentary elderly subjects (Safdar et al., 2010). Thus, targeting Nrf2 may be an effective therapeutic treatment for aged muscle atrophy. Our findings demonstrate a mechanistic link between RC and Nrf2 and suggest RC as a new Nrf2 activator.

Taken together, RC and its active compound, rutin, ameliorated muscle atrophy and helped in maintaining redox homeostasis. Therefore, rutin-rich RC could be a promising herbal medicine that is beneficial for muscle health.

## Data Availability

The datasets presented in this study can be found in online repositories. The names of the repository/repositories and accession number(s) can be found in the article/Supplementary Material.
