# Supplementary material for: Castor Oil Plant (Ricinus communis L.) Leaves Improve Dexamethasone-Induced Muscle Atrophy via Nrf2 Activation
Source: Front Pharmacol. 2022 Jul 5;13:891762. doi: 10.3389/fphar.2022.891762 (PMC9294160; doi:10.3389/fphar.2022.891762)
Supplement: Supplementary file 1 [file Table1.DOCX]

**Supplemental Data**

**Castor oil plant (*Ricinus communis* L.) leaves improve dexamethasone-induced muscle atrophy via Nrf2 activation**

**Hyunjung Lee^1#^, Young In Kim^1,2#^, Min Jung Kim^3^, Jeong-Hoon Hahm^1^, Hyo Deok Seo^1^, Tae Youl Ha^1,4^, Chang Hwa Jung^1,4^, and Jiyun Ahn^1,4*^**

^1^Aging and Metabolism Research Group, Korea Food Research Institute, Wanju-gun, South Korea

^2^Department of Food Science and Technology, Jeonbuk National University, Jeonju-si, South Korea

^3^Healthcare Research Group, Korea Food Research Institute, Wanju-gun, Republic of Korea

^4^Department of Food Biotechnology, University of Science and Technology, Daejeon-si, South Korea

**Supplementary Table 1. Primer sequences for RT-PCR**

| **Gene** | **Primer Sequences** | |
| --- | --- | --- |
| Atrogin-1 | Forward  Reverse | 5'- AAGGCTGTTGGAGCTGATAGCA -3'  5'- CACCCACATGTTAATGTTGCCC -3' |
| MuRF1 | Forward  Reverse | 5'- TGTCTCACGTGTGAGGTGCCTA -3'  5'- CACCAGCATGGAGATGCAGTTAC -3' |
| Myostatin | Forward  Reverse | 5'- ACG CTA CCA CGG AAA CAA TC -3'  5'- GGA GTC TTG ACG GGT CTG AG -3' |
| MyoG | Forward  Reverse | 5'-GTAGTAGGCGGTGTCGTAGC -3'  5'- CCACGATGGACGTAAGGGAG -3' |
| MyoD | Forward  Reverse | 5'- CCGTGTTTCGACTCACCAGA -3'  5'- GTAGTAGGCGGTGTCGTAGC -3' |
| NOX1 | Forward  Reverse | 5'- GGTTGGGGCTGAACATTTTTC -3'  5'- TCGACACACAGGAATCAGGAT -3' |
| NOX2 | Forward  Reverse | 5'- TGAAGAAAACCCCTTGTGCT -3'  5'- TTCTGTGCTGTCCCAGTGAG -3' |
| NOX4 | Forward  Reverse | 5'- GAAGGGGTTAAACACCTCTGC -3'  5'- ATGCTCTGCTTAAACACAATCCT -3' |
| MHCⅠ | Forward  Reverse | 5'- CTCAAGCTGCTCAGCAATCTATTT -3'  5'- GGAGCGCAAGTTTGTCATAAGT -3' |
| MHCⅡα | Forward  Reverse | 5'- AAGCGAAGAGTAAGGCTGTC -3'  5'- GTGATTGCTTGCAAAGGAAC -3' |
| MHCⅡβ | Forward  Reverse | 5'- CACCTGGAGCGGATGAAGAAGAAC-3'  5'- GTCCTGCAGCCTCAGCACGTT -3' |
| Cylophilin | Forward  Reverse | 5'- TGGAGAGCACCAAGACAGACA -3'  5'- TAGTAACAGCTGAGGCCGT -3' |
| 18s | Forward  Reverse | 5'- CTCAACACGGGA AACCTCAC -3'  5'- CGCTCCACCAACTAAGAACG -3' |

**Supplementary Table 2. Antibody for Western Blot**

| **Antisera** | **Source** | | **Dilution** |
| --- | --- | --- | --- |
| T-MHC | DSHB | | 1:1000 |
| MHCⅠ | DSHB | | 1:1000 |
| MHCⅡα | DSHB | | 1:1000 |
| MHCⅡβ | DSHB | | 1:1000 |
| Atrogin-1(Fbx32) | Abcam | | 1:1000 |
| MuRF1 | Abcam | | 1:1000 |
| Puromycin | Millipore | | 1:1000 |
| Phospho-mTOR (Ser2448) | Cell Signaling | | 1:1000 |
| mTOR | Cell Signaling | | 1:1000 |
| Phospho-4E-BP1 (Thr37/46) | Cell Signaling | | 1:1000 |
| 4E-BP1 | Cell Signaling | | 1:1000 |
| Phospho-AMPKα | Cell Signaling | | 1:1000 |
| AMPKα | Cell Signaling | | 1:1000 |
| Phospho-Akt (Ser473) | Cell Signaling | | 1:1000 |
| Akt | Cell Signaling | | 1:1000 |
| Phospho-p70 S6 Kinase (Thr389) | Cell Signaling | | 1:1000 |
| p70 S6 Kinase | Abcam | | 1:1000 |
| Phospho-FoxO3a (Ser294) | Cell Signaling | | 1:1000 |
| FoxO3a | Cell Signaling | | 1:1000 |
| Glucocorticoid Receptor | Cell Signaling | 1:1000 | |
| Phospho-PI3Kinase p85 | Cell Signaling | 1:1000 | |
| PI3Kinase p85 | Cell Signaling | 1:1000 | |
| Phospho-AKT | Cell Signaling | 1:1000 | |
| AKT | Cell Signaling | 1:1000 | |
| Phospho-p38 | Cell Signaling | 1:1000 | |
| p38 | Cell Signaling | 1:1000 | |
| Phospho-p42/44 MAPK | Cell Signaling | 1:1000 | |
| p42/44 MAPK | Cell Signaling | 1:1000 | |
| **Antisera** | **Source** | | **Dilution** |
| Nrf2 | Novus | 1:1000 | |
| GCLC | Abcam | 1:1000 | |
| HMOX1 | Novus | 1:1000 | |
| HO-1 | Novus | 1:1000 | |
| NQO1 | Novus | 1:1000 | |
| LaminB | Invitrogen | 1:2000 | |
| beta actin | Santa Cruz | 1:2000 | |
| GAPDH | Santa Cruz | 1:2000 | |

**
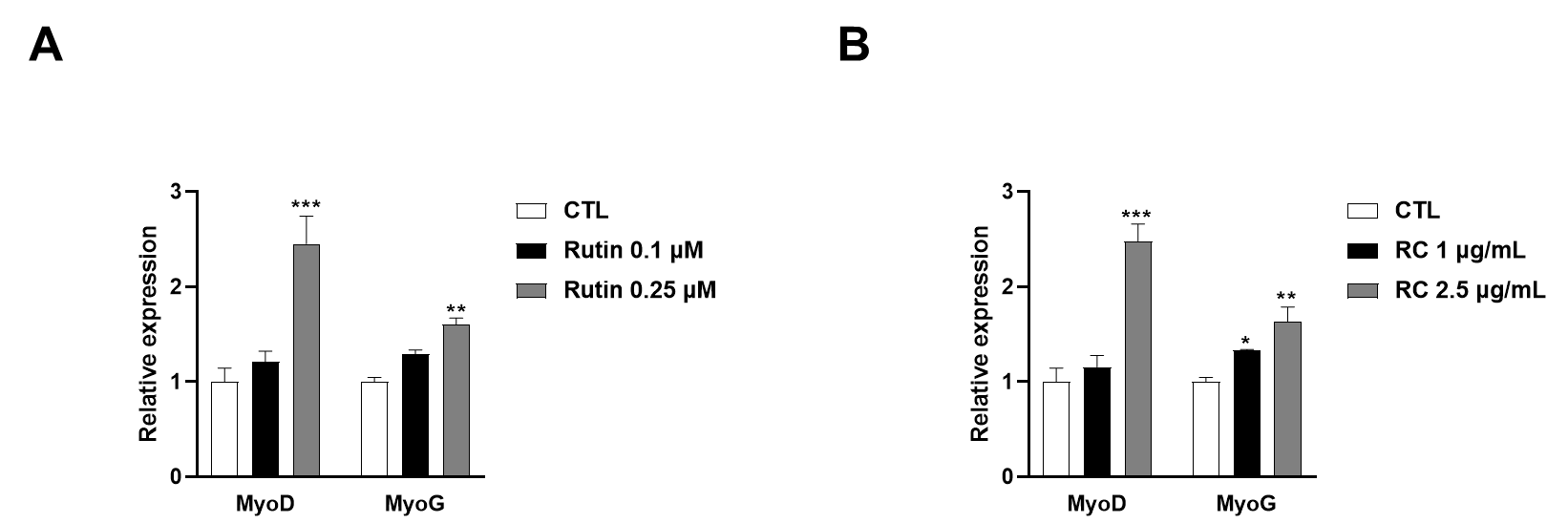
**

**Supplementary Figure 1. Effects of rutin (A) and RC (B) on the expressions of myogenic markers.**

**
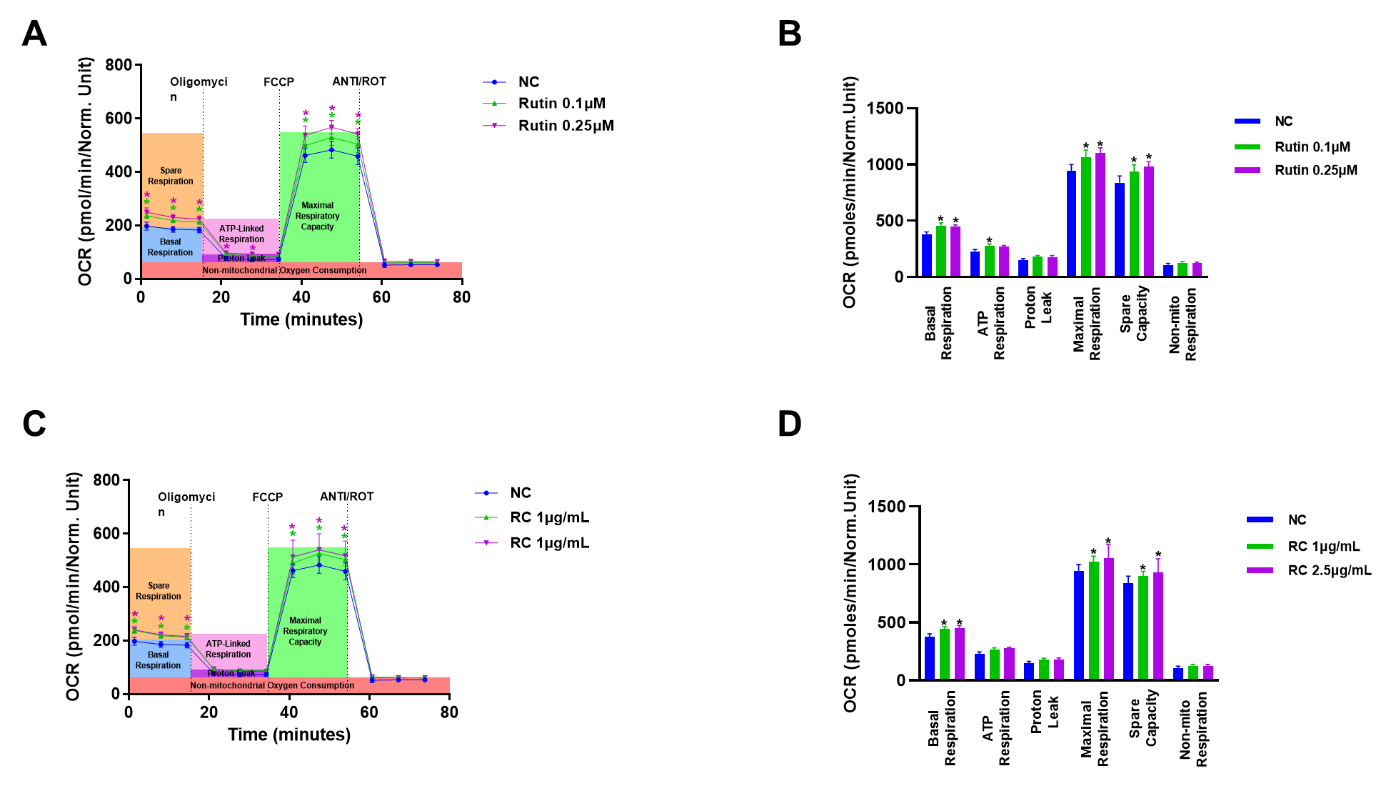
**

**Supplementary Figure 2. Effects of rutin and RC on the mitochondrial OCR (A and C) and mitochondrial respiration (B and D) in C2C12 myoblasts. The OCR was normalized to cell number of each well.**
